# Analysis of the virulence, infection process, and extracellular enzyme activities of *Aspergillus nomius* against the Asian corn borer, *Ostrinia furnacalis* guenée (Lepidoptera: Crambidae)

**DOI:** 10.1080/21505594.2023.2265108

**Published:** 2023-11-09

**Authors:** Xiaowu Wang, Xinhua Ding, Zihan Yuan, Zunzun Jia, Kaiyun Fu, Faqiang Zhan, Wenchao Guo, Liuyan Zhou, Haiqiang Li, Jinping Dai, Zhifang Wang, Yuqing Xie, Xinping Yang

**Affiliations:** a Institute of Microbiology Applications, Xinjiang Academy of Agricultural Sciences, Ürümqi, PR China; b Key Laboratory of Integrated Pest Management on Crops in Northwestern Oasis, Ministry of Agriculture, Ürümqi, PR China; c Institute of Plant Protection, Xinjiang Academy of Agricultural Sciences, Ürümqi, PR China

**Keywords:** *Aspergillus nomius*, *ostrinia furnacalis*, lethal efficacy, infection processes, cuticle, extracellular enzymes activity

## Abstract

The control of *Ostrinia furnacalis*, a major pest of maize in Xinjiang, is challenging owing to the occurrence of resistant individuals. Entomopathogenic fungi (EPF) are natural insect regulators used as substitutes for synthetic chemical insecticides. The fungus *Aspergillus nomius* is highly pathogenic to *O. furnacalis*; however, its virulence characteristics have not been identified. This study aimed to analyse the lethal efficacy, mode of infection on the cuticle, and extracellular enzyme activity of *A. nomius* against *O. furnacalis*. We found that the mortality and mycosis of *O. furnacalis* were dose-dependent when exposed to *A. nomius* and varied at different life stages. The egg-hatching and adult emergence rates decreased with an increase in conidial suspension. The highest mortality (83.33%, 7 d post-infection [DPI]) and mycosis (74.33%, 7 DPI) and the lowest mortality response (8.52 × 10^3^ conidia mL^−1^) and median lethal time (4.91 d) occurred in the 3^rd^ instar larvae of *O. furnacalis*. Scanning electron microscopy indicated that numerous conidia germination and infection structure formation may have contributed to the high pathogenicity of *A. nomius* against *O. furnacalis*. There were significant correlations between *O. furnacalis* mortality and the activities of extracellular protease, lipase, and chitinase of *A. nomius*. This study revealed the infection process of the highly pathogenic *A. nomius* against *O. furnacalis*, providing a theoretical basis and reference for strain improvement and field application of EPF.

## Graphical abstract

**Figure:**
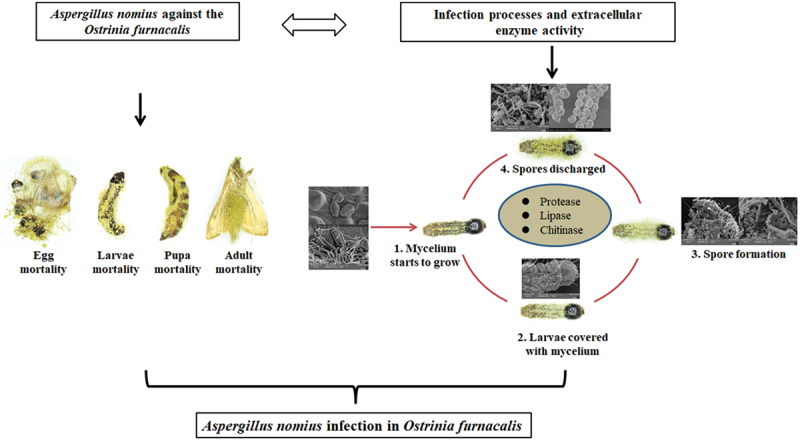

## Introduction

The Asian corn borer, *Ostrinia furnacalis* (Guenée) (Lepidoptera: Crambidae), particularly during the larval stage, is an important pest of maize (*Zea mays* L.) in China [1,2]. It poses a serious threat to maize production and causes substantial economic losses owing to direct damage and transmission of several plant pathogens, such as *Fusarium* spp. and *Ustilago maydis* [3,4]. Currently, the primary strategy for managing this insect pest in Xinjiang, China, is applying novel diamide insecticides to control *O. furnacalis* populations; however, this strategy is limited by the occurrence of resistant individuals [2]. Moreover, insecticides have undesirable side effects; therefore, the development of biological control methods is more advantageous for preventing and controlling agricultural pests than insecticides [5].

Entomopathogenic fungi (EPF) are an environmentally friendly alternative to chemical insecticides and are important natural regulators of pest insect populations [6]. Among the several groups of biocontrol agents used against insect pests, the most promising mitosporic fungi include *Beauveria bassiana*, *Metarhizium anisopliae*, *Aspergillus nomius*, *Lecanicillium lecanii*, and *Isaria fumosorosea* [2]. Of these, the genus *Beauveria* has been commercially mass-produced and successfully used to control *O. furnacalis* and related insects in greenhouses in China [7–9]. However, increasing evidence shows that the virulence of EPF is often associated with their high genetic diversity, environmental and host adaptations, and the extent of their additional non-target effects [10–13]. Therefore, the application of local fungal strains, instead of exotic strains, to control insects is a promising strategy [2,14], suggesting the effective use of local fungal strains for the biological control of insect pests. However, EPF virulence differs depending on the instar stages of the insect host [13,15,16].

Unlike viruses and bacteria, EPF possesses the unique ability to infect their host directly through the integument [17]; therefore, they are potent alternatives for insect management [2]. The mode of action of EPF against host insects begins with the attachment of the conidia to the insect integument, followed by germination, formation of an infection structure, and subsequent penetration of the cuticle. The hemocoel is then colonized, and the insect is eventually killed. Sporulation occurs after the emergence of the fungus from a mycosed cadaver [18]. Extracellular enzymes, including chitinase, protease, and lipase, which facilitate infection, are also produced by EPF when the insect’s integument is penetrated [19]. Therefore, knowledge of the infection processes and extracellular enzyme activity of EPF is essential to determine its potential for biocontrol [20–24]. Because EPF virulence can differ considerably according to the developmental stage of the pest [25], it is essential to obtain knowledge on the effectiveness of EPF in all life stages of an insect to comprehensively evaluate its regulatory effect on insect pests [26].

We have previously identified and isolated an EPF, *A. nomius* ACB1041, from a cadaver of *O. furnacalis* during a maise field investigation in Xinjiang from March-May 2019 and 2020 [2]. Several studies have reported the potential of *Aspergillus* spp., viz. *A. nomius*, *A. flavus*, *A. kanagawaensis*, as a biocontrol agent to suppress pest populations, including those that threaten stored products and public health [2,17,27–29], such as *Dolichoderus thoracicus* [30], mosquitoes [17], *Diaphorina citris* [29], *Spodoptera litura* [28,31], *Oligonychus coffeae* [32], *Anopheles gambiae*, and *Culex quinquefasciatus* [33]. Although the insecticidal activity of some *Aspergillus* species may be attributed to the secretion of mycotoxins (e.g. aflatoxins, ochratoxins, fumonisins, zearalenone and various phenolic compounds viz. gallic acid, caffeic acid) [34], the mycotoxins also showed its safety for mammals, as evidenced by negligible toxicity on rats [28]. However, the mode of action of *Aspergillus* spp. against insects has not yet been reported. There is limited knowledge on the virulence of *A. nomius* in Xinjiang against the different instar stages of *O. furnacalis*. In addition, the mode of action and extracellular enzymes of *A. nomius* remain unknown, although its pathogenicity against insects, such as *O. furnacalis*, has been reported [2,19].

This study aimed to (i) evaluate the virulence of *A. nomius* against *O. furnacalis* at different life stages; (ii) use optical and scanning electron microscopy to observe the infection process on the *O. furnacalis*, which are highly susceptible to *A. nomius*; and (iii) study the potential of *A. nomius* to produce extracellular enzymes. Moreover, this study aimed to elucidate the mechanism involved in *A. nomius* infection and evaluate its efficacy in suppressing the *O. furnacalis* population. This highly pathogenic strain was eventually developed into an effective biopesticide for the eco-friendly management of *O. furnacalis*. Our findings provide a better understanding of the mode of action of *A. nomius* infection and reveal how *A. nomius* succeeds in infecting *O. furnacalis*.

## Materials and methods

### Fungus and culture conditions

For this study, *A. nomius* ACB1041 (registered as the benA and CaM sequence of *A. nomius*) was originally isolated from the cadavers of naturally infected *O. furnacalis* overwintering larvae [2] and deposited in GenBank under Accession nos. OK625287 and OK625286. The fungal strain was also maintained at the China Center for Type Culture Collection (CCTCC), Wuhan, China, under the Accession number M 20,211,535 ACB1041.4. The strain was cultured on Sabouraud dextrose agar (SDA) and incubated at 26 °C for 15 d under a 16:8 h (light: dark) photoperiod. Conidia were harvested from 15-d-old SDA sporulated cultures [2] and suspended in 10 mL 0.05% Tween 80 solution in universal bottles containing 6‒8 glass beads (3-mm diameter). To break the conidial clumps and ensure a homogeneous suspension, the suspension was vortexed for 5 min at approximately 700 rpm [35].

### Preparation of the conidial suspension

For the virulence bioassay, conidial suspensions were adjusted to eight strain concentrations ranging from 1.0 × 10^1^ conidial mL^−1^ to 1.0 × 10^8^ conidial mL^−1^ through dilution in a Neubauer chamber under a light microscope. Before the experiments, conidia viability tests were conducted according to the method of Akutse et al. [27,35].

### Insect rearing

A laboratory colony of *O. furnacalis* was established using overwintering larvae collected from maize crops from Yining County in Yining City, Xinjiang Uygur Autonomous Region, China (81°52’ E, 43°98’ N) from January to March 2019.

The laboratory colony of *O. furnacalis* larvae was reared on a modified artificial diet [36] until pupation under controlled conditions at 27 ± 1 °C, 70–80% relative humidity (RH), and under a 16:8 h (L:D) photoperiod [2]. The pupae were placed in ventilated mating cages (25 × 25 × 40 cm with sulphate paper on the sides and top) until adult emergence. The newly emerged adults were paired, kept in a ventilated mating cage, and fed a 10% sugar solution [2,36]. Sulphate paper was removed 24 h after oviposition, and pupae were transferred to moistened plastic boxes until hatching, following the approach used by Guo et al. [35,37]. The newly hatched neonates to the 2^nd^ instar larvae were reared in the same group and then placed in plastic containers (15 × 7 × 5 cm) lined with sterile moistened filter paper to absorb excess moisture. After moulting to the 3^rd^ instar larvae stage, the larvae were individually fed in 12-well culture plates containing fresh modified artificial diet blocks. The larval instars were identified by their head width using a previously described method [38].

### Dose-response bioassay of A. nomius ACB1041 against different stages of life of O. furnacalis

#### Against different instar stages of O. furnacalis larvae

Before the dose-response bioassays, the different life stages of *O. furnacalis* (egg, larvae from the 1^st^ to the 5^th^ instar larvae, pupae, and adults) were identified within the colony described above. A dose-response bioassay of all *O. furnacalis* life stages was conducted. Similar-sized pieces (approximately 5 × 5 × 6 mm) of fresh but modified artificial food were prepared, and one piece was placed in each well of a 12-well cell culture plate. The *O. furnacalis* were transferred using a camel hairbrush onto the modified artificial diet piece in each well of the 12-well cell culture plates and allowed to settle. Individual larvae were placed in each separate well. Three cell culture plates represented a replicate (*n* = 36), and three were used for each conidial suspension concentration (*n* = 108 insects per treatment). The modified cut food and larvae within the cell culture plates were then sprayed with 3 mL spore suspension using a Potter spray tower PDE0012 (Burkard, Burkard Scientific, UK). Before spraying, a sterilised paper towel was placed under the food to absorb any excess fungal suspension. Treatments were applied from the lowest to the highest concentration to prevent cross-contamination. The spray tower was washed twice with 75% alcohol after each application and once with 0.05% (v/v) Tween 80 between the application of different concentrations. The control group was sprayed with 0.05% Tween 80. All experiments were repeated three times.

Treatment and control groups were placed separately in a chamber under controlled conditions (27 ± 1 °C, 70% ± 5% RH, 12 h light: dark photoperiod) [2]. Mortality, mycosis, and larval development were monitored daily for 15 d until all individuals had died or a new *O. furnacalis* instar had emerged [39]. Mycosis was confirmed based on the method used in our previous research [2]. The mortality rate was corrected using the number of larvae that exhibited natural death/paralysis in the 0.5% Tween 80 control according to the Schneider – Orelli formula [40].

#### Against O. furnacalis pupae

*O*. *furnacalis* pupae were placed in Petri dishes (90-mm diameter) (lined with sterilised moistened filter paper to absorb excess spore suspension) and then treated with 3 mL of spore suspension using a Burkard Potter. The inoculated pupae (30) were treated as one replicate, and three replicates were used for each conidial suspension concentration (*n* = 90 insects per treatment). The controls were treated with 0.05% Tween 80. After treatment, pupae were transferred into Petri dishes at 26 ± 1 °C and 70%–80% RH, and each treatment was replicated thrice. The emergence rate and mortality were monitored daily for 15 d until all individuals had died or adults had emerged, and mycosis was confirmed by the presence of hyphae and conidia on the surface of the cadaver using the method reported in our previous research [2].

#### Against O. furnacalis adult

A total of 15 pairs of 2-d-old newly emerged adults were placed in a 200-mL beaker with a 10-mesh nylon sieve (2 mm) and then treated with 3 mL of spore suspension using a Burkard Potter. The controls were treated with 0.05% Tween 80 [30]. The inoculated adults were then placed separately in mating cages (25 × 25 × 40 cm), and the moths were fed a 5% honey solution. The mating cages were maintained in a rearing room at 26 ± 1 °C and 70–80% RH. Mortality was monitored daily for 15 d until all individuals had died, and mycosis was confirmed based on the method used in our previous research [2].

#### Against O. furnacalis eggs and neonates

Fresh sulphate paper containing similar-sized *O. furnacalis* eggs (24 h) was cut into pieces measuring approximately 0.5 × 0.5 cm, and each piece was placed in a Petri dish (90-mm diameter) lined with sterile moistened filter paper. The paper and eggs were treated with 3 mL of spore suspension using a Burkard Potter, and the controls were treated with 0.05% Tween 80. After treatment, the paper and eggs were air dried for 30 min on a clean bench (25 ± 2 °C) and then transferred into Petri dishes and incubated at 26 ± 1 °C and 70–80% RH. Each treatment was replicated three times. The hatched larvae and egg mortality numbers were recorded daily for 15 d until all individuals died or neonate larvae emerged.

The neonate larvae that emerged from the *O. furnacalis* eggs on each piece of treated sulphate paper were counted, fed with fresh modified artificial diet blocks (approximately 5 × 5 × 6 mm) in clear plastic containers (15 × 7 × 5 cm) lined with sterile moistened filter paper, incubated at 26 ± 1 °C, and monitored daily. Mortality of the neonate larvae due to fungal infection was recorded daily for 15 d post-emergence. Mycosis was also evaluated for dead neonate larvae, as described above, and mycosis was confirmed using the method reported in our previous research [2]. All treatments were replicated three times.

### Optical microscopy (OM) and scanning electron microscopy (SEM)

#### OM observations

A Nikon Eclipse 80i OM (Nikon, Nikon Instruments Inc., Tokyo, Japan) was used to capture photographs of the immersed different life stages of *O. furnacalis* and observe the morphological characteristics of *O. furnacalis* following inoculation with the *A. nomius* ACB1041 strain from days 1‒15 post-inoculation. In each studied time interval and different life stages, five *O. furnacalis* were selected randomly, representing five replicates used to observe the morphological characteristics of *O. furnacalis* by the *A. nomius* ACB1041 infection process.

#### SEM observations

To document the epidermal infection process of ACB1041 strains, specimens were collected at 4, 8, 12, 24, 48, 72, 96, 120, and 144 h post-inoculation and fixed with 2.5% glutaraldehyde solution (Bodi Chemical Co., Ltd., Tianjin, China) at 4 °C for 12 h. The specimens were then dehydrated for 10 min each in a series of ascending order prepared the serial dilutions of ethanol (30, 50, 70, 80, and 100%), were dipped in isoamyl acetate for 30 min, and dried using a critical-point drying apparatus using carbon dioxide (Eiko XD-1). Finally, the samples were sputter-coated with gold and observed under a VEGA3 tungsten SEM (TESCAN, Brno, Czech).

### Estimation of extracellular enzyme activity of ACB1041 strain

#### Liquid culture for enzyme production of ACB1041 strain

To investigate the extracellular enzyme activity of the ACB1041 strain during the epidermal infection process of *O. furnacalis*, a liquid medium was prepared containing 0.02% KH_2_PO_4_, 0.01% CaCl_2_, 0.01% MgSO_4_, 0.02% Na_2_HPO_4_, 0.01% ZnCl_2_, 0.01% yeast extract (Merck & Co., Rahway, NJ, USA), and 5% (weight) of larval cuticle; this was then sterilised in an autoclave (LDZX-75KB) at 121 °C for 30 min. Before preparing the liquid medium, the cuticles of *O. furnacalis* were obtained according to Velavan et al. [24,29]. The liquid medium was then inoculated in a flask with an ACB1041 isolate concentration of 3 mL of 10^8^ conidia/mL, repeated three times. The flask was maintained on a 125 rpm cryogenic shaker (MAXQ5000) at 25 ± 1 °C. Subsequently, 1 mL was harvested from the culture flask daily for 15 d, placed in a 1.5-mL EP tube, and centrifuged (Eppendorf-5804 R) at 4 °C and 12,000 rpm for 15 min to obtain the supernatant for the enzyme assay.

#### Extracellular enzyme activities

Lipase, chitinase, and protease activities were determined based on the manufacturer’s instructions (Grace Biotechnology Co., Ltd., Suzhou, China). The lipase enzyme reaction mixture comprised 10 μL of fresh enzyme sample supernatant, 40 μL of Reagent I, and 150 μL of Reagent II placed in a 1.5-mL EP tube (with six replicates). After homogenization, a 200 μL mixture was added to 96-well cell culture plates (200 μL per well cell) (Grace Biotechnology Co., Ltd., Suzhou, China). Absorbance was recorded at 405 nm (A1) and then after 10 min at 405 nm (A2), where ΔA = A2 − A1.

The chitinase enzyme reaction mixture of the test group within each 1.5-mL EP tube comprised 80 μL of fresh enzyme sample supernatant, 80 μL of Reagent I, and 100 μL of Reagent II. After homogenization, the mixture was incubated for 90 min before centrifugation at 4,000 rpm and 4 °C for 5 min. Subsequently, 150 μL of mixture supernatant was obtained, and 10 μL of Reagent III and 15 μL of Reagent Ⅳ were added to the supernatant in a new 1.5-mL EP tube. This was then homogenized, incubated for 60 min at 37 °C, and 50 μL of Reagent Ⅴ was added to the 1.5-mL EP tube. The mixture was homogenized and centrifuged at 4,000 rpm and 4 °C for 5 min to obtain 150 μL of supernatant. The 150 μL supernatant of the mixture was added to a new 1.5-mL EP tube with 200 μL of Reagent Ⅵ, homogenized, and then incubated for 10 min at 95–100 °C. Finally, 200 μL of the mixture was added to 96-well cell culture plates at 200 μL per well cell.

The composition of the chitinase enzyme reaction mixture of the control group was similar to that of the test group, except that 80 μL of fresh enzyme sample supernatant was first incubated for 1 min at 100 °C before adding Reagents I and II to the 1.5-mL EP tube. The method used was then identical to that of the test group and followed the manufacturer’s instructions. The absorbance of both groups was recorded as A control group and A test group at 420 nm, and ΔA= A_control group_ - A_test group_.

The protease enzyme reaction mixture of the test group comprised 500 μL of fresh enzyme sample supernatant, 500 μL of Reagent I, and 500 μL of Reagent III in a 1.5-mL EP tube. After homogenization and incubation for 10 min, the mixture was centrifuged at 1,500 rpm and 4 °C for 10 min to obtain 250 μL of the supernatant of the mixture. This supernatant was added to a new 1.5-mL EP tube with 375 μL of Reagent Ⅳ and 250 μL Reagent Ⅴ, and the mixture was incubated for 20 min at 40 °C. Subsequently, 200 μL of the supernatant mixture was added to 96-well cell culture plates at 200 μL per well cell. Again, the procedure for the control group was similar to that of the test group and followed the manufacturer’s instructions; however, the remaining Reagent I was first incubated for 10 min at 40 °C before being added along with Reagents I and III to the 1.5-mL control EP tube. Finally, absorbance was recorded as A test group and A control group at 680 nm, and ΔA= A_test group_ - A_control group_. All bioassays described above were repeated six times.

### Statistical analysis

The effect of the spore concentration of *A. nomius* ACB1041 on different life stages of *O. furnacalis* was compared using the “chi-squared test” functions in the “contingency table analyses” program of Graph Pad Prism 8.0. A survival analysis was conducted using the “survival curve” functions in the “survival analysis” program of Graph Pad Prism 8.0. The median lethal concentration (LC_50_) and median lethal time (LT_50_) were determined by conducting a probit analysis using IBM SPSS Statistics software (version 20.0.). The data of LC_50_ and LT_50_ values were subjected to analysis of variance (ANOVA), and differences among treatments were compared using the Tukey’s test (*P* > 0.05) to determine significance using IBM SPSS Statistics software (version 20.0.). The relationships between mortality and concentration and extracellular enzyme activities were analysed using the “linear regression” and “Fit spline/LOWESS” functions, respectively, in the “XY analyses” program of Graph Pad Prism 8.0. The results were considered statistically significant when *p <* 0.05. Data were analysed using IBM SPSS Statistics software (version 20.0.), and figures were constructed using Graph Pad Prism 8.0 (Graph Pad Prism).

## Results

### Virulence bioassay of A. nomius against different life stages of O. furnacalis

**Figure 1.**
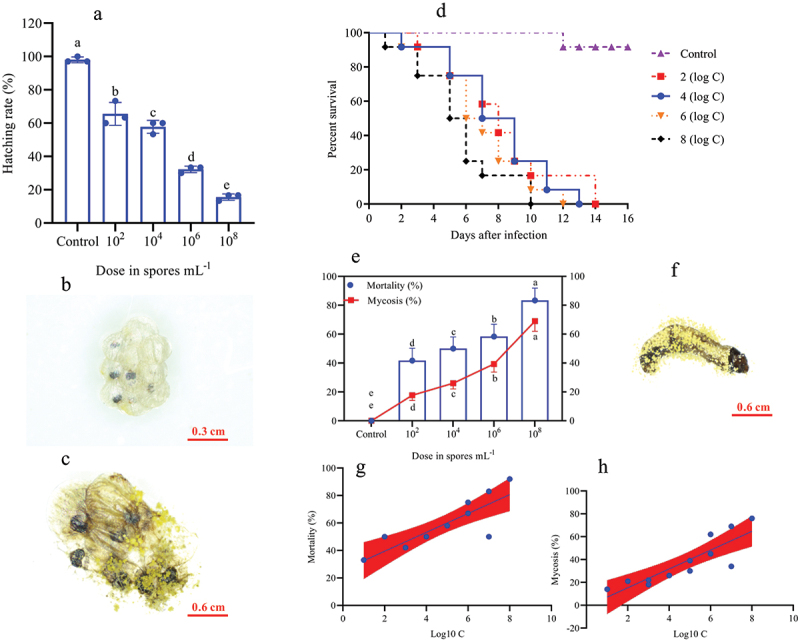
Effect of *O. furnacalis* eggs on the hatching rate and the survival, mortality, and mycosis of the 1^st^ instar larvae of *O. furnacalis* (*n* = 90) following spray infection with different *A. nomius* concentrations. (a) hatching rate of *O. furnacalis* eggs at 7 d post-infection (DPI) with *A. nomius*; (b and c) healthy eggs and mycosis of eggs; (d) survival probability of the 1^st^ instar larvae of *O. furnacalis*; (e) mortality and mycosis of the 1^st^ instar larvae of *O. furnacalis* infected with*A. nomius*; (f) *A. nomius*mycosis of the 1^st^ instar larvae of *O. furnacalis*; (g and h) log-probit regression line of concentration-mortality and mycosis of the 1^st^ instar larvae of *O. furnacalis*to *A. nomius.*

*O*. *furnacalis* eggs were successfully infected with *A. nomius* ACB1041 at all spore concentrations; a significant effect was observed between the concentration and ovicidal response of the eggs (χ^2^ = 162.8, df = 7, *p =* 0.0150). The *A. nomius* concentration invoked a strong and increasing ovicidal response (15.56%, hatching rate) in 10^8^ conidia/mL, followed by 10^6^ conidia/mL (32.22%) and 10^4^ conidia/mL (57.78%); in contrast, 10^2^ conidia/mL provoked a weak response (65.56%) (Figure 1a). Infection with *A. nomius* was confirmed when the fungus sporulated, and the eggs did not hatch (Figure 1b).

**Figure 2.**
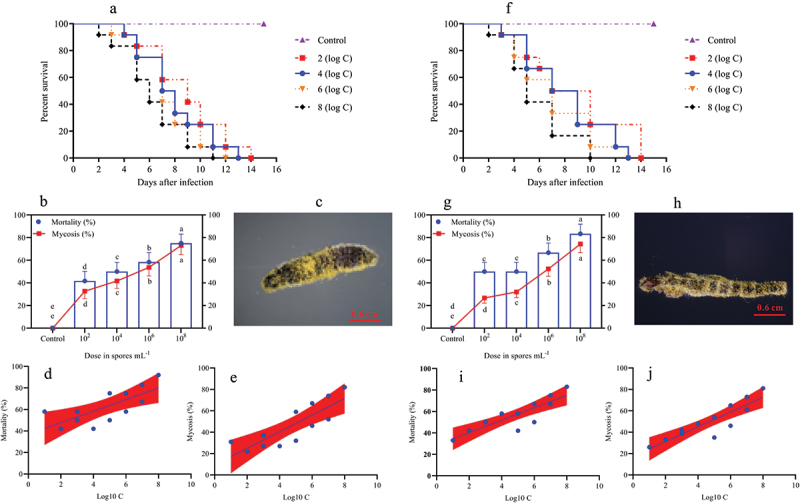
Survival, mortality, and mycosis of the 2nd and 3rd instar larvae of O. furnacalis (*n* = 90) following spray infection with different concentrations of A. nomius. (a and e) survival probability of the (a) 2nd and (e) 3rd instar larvae of O. furnacalis; (b and g) mortality and mycosis of the (b) 2nd and (g) 3rd instar larvae of O. furnacalis infected with A. nomius; (c and h) A. nomius mycosis on the (c) 2nd and (h) 3rd instar larvae of O. furnacalis; (d, e, i, and j) log-probit regression line of (d and i) concentration-mortality and (e and j) mycosis response of the 2nd and 3rd instar larvae of O. furnacalis to A. nomius.

**Figure 3.**
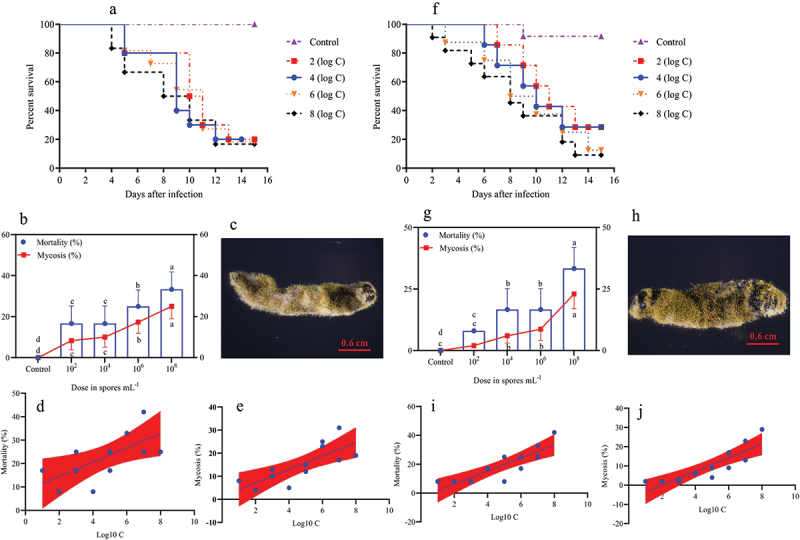
Survival, mortality, and mycosis of the 4^th^ and 5^th^ instar larvae of *O. furnacalis* (*n* = 90) following spray infection with different *A. nomius* concentrations. (a and f) survival probability of the (a) 4^th^ and (f) 5^th^ instar larvae of *O. furnacalis*; (b and g) mortality and mycosis of the (b) 4^th^ and (g) 5^th^ instar larvae of *O. furnacalis* infected with *A. nomius*; (c and h) *A. nomius* mycosis of the (c) 4^th^ and (h) 5^th^ instar larvae of *O. furnacalis*; (d, e, i, and j) log-probit regression line of (d and i) concentration-mortality and (e and j) mycosis response of 4^th^ and 5^th^ instar larvae of *O. furnacalis* to *A. nomius*.

**Figure 4.**
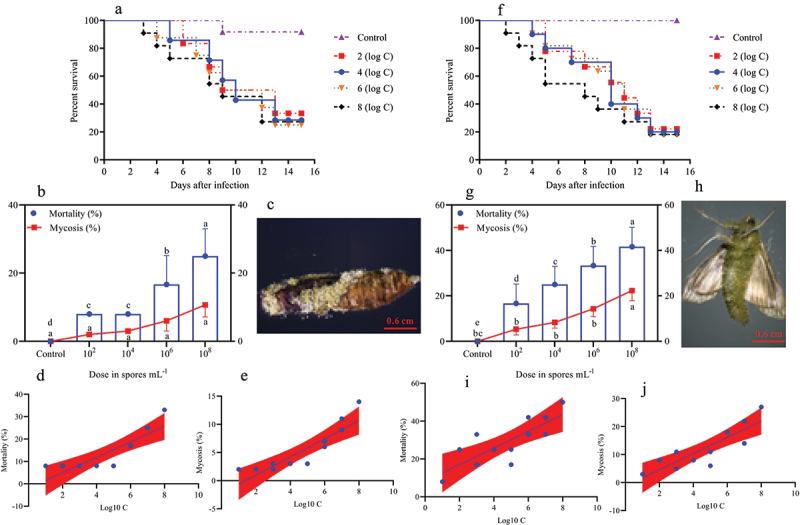
Survival, mortality, and mycosis of pupae and adults of *O. furnacalis* (*n* = 90) following spray infection with different *A. nomius* concentrations. (a and f) survival probability of *O. furnacalis* (a) pupae and (f) adults; (b and g) mortality and mycosis of *O. furnacalis* (b) pupae and (g) adults infected with *A. nomius*; (c and h) mycosis of *A. nomius* on *O. furnacalis* (c) pupae and (h) adults; (d, e, i, and j) log-probit regression line of (d and i) concentration-mortality and (e and j) mycosis response of *O. furnacalis*pupae and adult to *A. nomius.*

In the larval stage, the mortality and mycosis frequency increase at different spore concentrations was dose-dependent. The survival probability test indicated that the survival rates of *O. furnacalis* at the 1^st^ (Figure 1d, Log-rank test: χ^2^ = 31.13, df = 7, *p =* 0.0001), 2^nd^ (Figure 2a, Log-rank test: χ^2^ = 30.59, df = 7, *p =* 0.0001), 3^rd^ (Figure 2f, Log-rank test: χ^2^ = 32.04, df = 7, *p =* 0.0001), 4^th^ (Figure 3a, Log-rank test: χ^2^ = 17.64, df = 7, *p =* 0.0015), and 5^th^ (Figure 3f, Log-rank test: χ^2^ = 16.81, df = 7, *p =* 0.0021) instar larvae were significantly lower when exposed to higher *A. nomius* concentrations. Furthermore, *O. furnacalis* mortality occurred earlier when exposed to higher concentrations (Figure 1f, 2 a, 2 f, 3 a, 3 f, 4a, and 4f) and increased with an increase in spore concentration (Figure 1e, 2b, 2g, 3b, 3g). All 1^st^, 2^nd^, and 3^rd^ *O. furnacalis* instar larvae had died by 11 d post-infection (DPI) (Figs. Figure 1d, 2a–f), whereas the survival rates of 4^th^ and 5^th^ instar larvae were 33.33% (Figure 3) and 36.36% (Figure 3f) at 1.0 × 10^8^ conidia mL^−1^, respectively. Similarly, mycosis also increased with increased spore concentration (Figure 1e, 2 b, 2 g, 3b, and 3b). Furthermore, the mortality and mycosis of 1^st^ (Figure 1g, F = 24.37, *p =* 0.0006, R^2^ = 0.7091; Figure 1h, F = 28.60, *p =* 0.0003, R^2^ = 0.7409), 2^nd^ (Figure 2d, F = 11.31, *p =* 0.0072, R^2^ = 0.5308; Figure 2e, F = 22.66, *p =* 0.0008, R^2^ = 0.6930), 3^rd^ (Figure 2i, F = 28.57, *p =* 0.0003, R^2^ = 0.7407; Figure 2j, F = 37.01, *p =* 0.0001, R^2^ = 0.7873), 4^th^ (Figure 3d, F = 7.555, *p =* 0.0205, R^2^ = 0.4304; Figure 3e, F = 14.24, *p =* 0.0036, R^2^ = 0.5875), and 5^th^ (Figure 3i, F = 27.26, *p =* 0.0004, R2 = 0.7316 and Figure 3j, F = 24.44, *p =* 0.0003, R2 = 0.7399) instar larvae were positively correlated with the spore concentrations of *A. nomius*.

**Figure 5.**
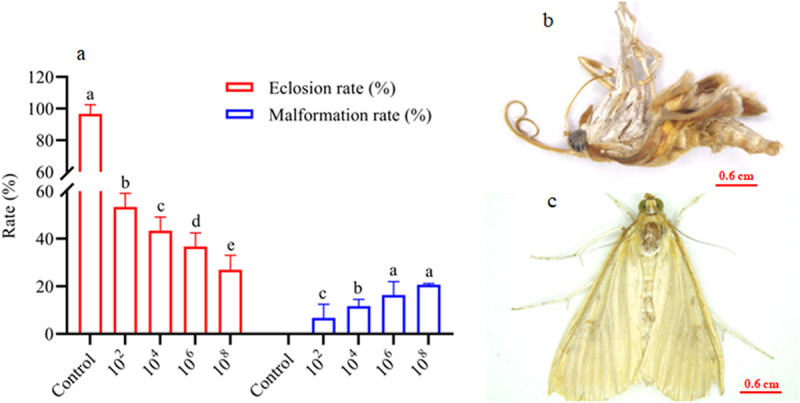
Effect of infection with *A. nomius* on the rate of pupa eclosion and pupa development of *O. furnacalis*.(a) pupa eclosion rate of *O. furnacalis* and malformed adults of*O. furnacalis* emerged in a group of pupae infected with different *A. nomius* concentrations; (b) malformed *O. furnacalis* adults; (c) healthy *O. furnacalis* adults.

In the pupae and adult stages, a similar trend of dose-dependent spore concentration was observed between *A. nomius* and mortality and mycosis (Figure 4). The mortality of pupae (Figure 4a; Log-rank test: χ^2^ = 10.96, df = 7, *p =* 0.0270) and adult *O. furnacalis* (Figure 4f; Log-rank test: χ^2^ = 17.57, df = 7, *p =* 0.0002) occurred earlier when exposed to higher concentrations, and a positive relation was observed between spore concentrations and mortality (Figure 4d–i) and mycosis (Figures 4e–j) of pupae and adults. Notably, several adults that emerged from the pupae infected with *A. nomius* were malformed (Figure 5b), and the malformation rate increased with an increase in the spore concentration (Figure 5a).

*Aspergillus nomius* infection was observed in all *O. furnacalis* life stages, and the maximum mortality and mycosis of early-stage larvae (including 1^st^, 2^nd^, and 3^rd^ instar larvae) exceeded 70% at 7 DPI. However, the late-stage larvae (including 4^th^ and 5^th^ instar larvae), pupae, and adults showed lower mortality and mycosis of less than 50% at 7 DPI, indicating that the early-stage larvae are more susceptible to *A. nomius*. Therefore, we selected early-stage *O. furnacalis* larvae for subsequent experiments on the pathogenicity of *A. nomius* toward *O. furnacalis* and determining the LC_50_ and LT_50_ values.

### LC_50_ and LT_50_ values of A. nomius

Significant differences in the LC_50_ and LT_50_ values of *A. nomius* across the different *O. furnacalis* life stages were identified based on dose and time mortality response studies (Tables 1 and 2). The LC_50_ values of *A. nomius* against *O. furnacalis* larvae from the 1^st^ to 5^th^ instar larvae, pupae, and adults were 1.42 × 10^5^, 2.17 × 10^5^, 8.52 × 10^3^, 2.30 × 10^8^, 2.19 × 10^8^, 8.20 × 10^7^, and 2.00 × 10^5^ conidia/mL, respectively. The LT_50_ values of *A. nomius* at 1.0 × 10^8^ spores/mL and 1.0 × 10^6^ spores/mL of the corresponding developmental stages were 3.90, 4.42, 4.91, 7.23, 7.16, 8.88, 6.35 d, and 5.72, 5.47, 6.05, 9.29, 11.16, 11.91, 8.83 d, respectively.

**Table 1.** LC_50_ values (conidia/mL) for *A. nomius* against different life-cycle stages of *O. furnacalis*..

Insect stage	χ^2^ †	LC50 ‡	LC_50_ confidence Interval		Concentration response (conidia/mL)					
					Test for slope			Test for intercept		
			Lower	Upper	Slope ± SE	*P*	95% CI	Intercept ± SE	*P*	95% CI
1^st^ instar	1.217	1.42 × 10^5^ b	1.37 × 10^3^	4.89 × 10^6^	0.67 ± 0.25	0.007	0.176–1.158	−3.44 ± 1.69	0.042	−5.125 to − 1.749
2^nd^ instar	0.495	2.17 × 10^5^ b	1.03 × 10^4^	8.80 × 10^6^	0.97 ± 0.36	0.006	0.267–1.676	−5.19 ± 2.22	0.019	−7.403 to − 2.971
3^rd^ instar	4.080	8.52 × 10^3^ a	6.03 × 10^2^	9.12 × 10^4^	0.24 ± 0.01	0.349	−0.262 to 0.741	−0.94 ± 2.70	0.727	−3.639 to 1.757
4^th^ instar	0.064	2.30 × 10^8^ d	1.68 × 10^7^	5.79 × 10^11^	0.45 ± 0.21	0.034	0.032–0.861	−3.73 ± 1.68	0.026	−5.413 to − 2.056
5^th^ instar	0.104	2.19 × 10^8^ d	1.21 × 10^7^	3.79 × 10^12^	0.24 ± 0.15	0.110	−0.055 to 0.545	−2.04 ± 1.37	0.136	−3.412 to − 0.672
Pupae	0.021	8.20 × 10^7^ c	5.34 × 10^5^	8.97 × 10^11^	0.11 ± 0.01	0.462	−0.188 to 0.414	−0.89 ± 2.22	0.687	−3.109 to 1.322
Adult	2.456	2.00 × 10^5^ b	1.54 × 10^3^	3.97 × 10^6^	0.13 ± 0.01	0.387	−0.166 to 0.428	−0.66 ± 2.19	0.764	−2.841 to 1.530

†Chi-squared; ‡LC_50_ of one treatment was significantly different if the lower and upper fiducial limits do not include the LC_50_ value of other treatments; the LC_50_ values are expressed as the concentration of conidia mL^−1^. Data on LC_50_ within a column followed by the same small letters were not significantly different (factorial ANOVA; Tukey’s test at *p* ≤0.05). LC_50_, median lethal concentration.

**Table 2.** LT_50_ values (days) of *A. nomius* against different life-cycle stages of *O. furnacalis*..

Insect stage	Chi-squared (χ^2^)	Concentration response (conidia/mL)								
		LT_50_	LT_50_ 95% CI		Test for slope			Test for intercept		
			Lower	Upper	Slope ± SE	*p*	95% CI	Intercept ± SE	*p*	95% CI
1^st^ instar	4.554	3.90 a	2.80	4.95	3.33 ± 0.73	0.000	1.894–4.775	−1.97 ± 0.53	0.000	−2.495 to − 1.443
2^nd^ instar	1.323	4.42 ab	3.53	5.33	5.01 ± 1.13	0.000	2.792–7.219	−3.23 ± 0.79	0.000	−4.026 to − 2.438
3^rd^ instar	2.264	4.91 b	4.04	5.80	4.58 ± 0.85	0.000	2.911–6.253	−3.17 ± 0.65	0.000	−3.814 to − 2.521
4^th^ instar	0.559	7.23 d	5.54	9.37	3.58 ± 1.01	0.000	1.603–5.561	−3.076 ± 0.89	0.001	−-3.964 to − 2.189
5^th^ instar	2.093	7.16 d	5.75	8.83	3.08 ± 0.57	0.000	1.959–4.196	−2.630 ± 0.513	0.000	−3.143 to − 2.188
Pupae	0.091	8.88 e	6.88	14.84	2.88 ± 0.83	0.001	1.257–4.499	−2.730 ± 0.704	0.000	−3.434 to − 2.026
Adult	1.605	6.35 c	5.06	8.04	3.03 ± 0.60	0.000	1.862–4.193	−2.43 ± 0.492	0.000	−2.922 to − 1.938

LT_50_, median lethal time; CI, confidence interval.

Data on LT_50_ within a column followed by the same small letters were not significantly different (factorial ANOVA; Tukey’s test at *p* ≤ 0.05).

### Ultramicroscopic changes during A. nomius infection in O. furnacalis

The *O. furnacalis* larvae mortality and mycosis rates and LC_50_ and LT_50_ ranking results showed that the 3^rd^ instar larvae of *O. furnacalis* were highly susceptible to infections with *A. nomius*. Therefore, the 3^rd^ instar larvae were selected for SEM analysis to determine how *A. nomius* successfully infected *O. furnacalis*.

**Figure 6.**
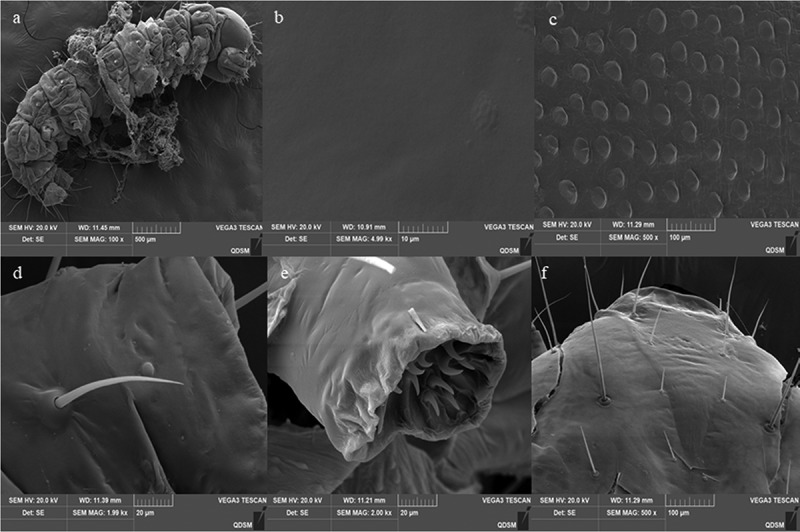
Cuticle topography of the 3^rd^ instar larvaeof*O. furnacalis*. (a) full view of a larva showing strumae and gentle surface topography; (b) gentle surface topography; (c) strumae surface topography; (d and e) sites beside the setae and proleg; (f) head capsule.

Surface topography analysis classified the *O. furnacalis* larval cuticle as having both a gentle and strumae surface topography (Figure 6). The strumae surface topography was covered with densely regular protuberances, including those in the central segments and on sites beside the setae and proleg (Figures. 6a and c). The gentle surface topography was flat and smooth with no protuberances (Figures 6b and 6–f), and the areas included the head capsule (Figure 6f), setal alveolus (Figure 6f), and proleg (Figures. 6d,e).

**Figure 7.**
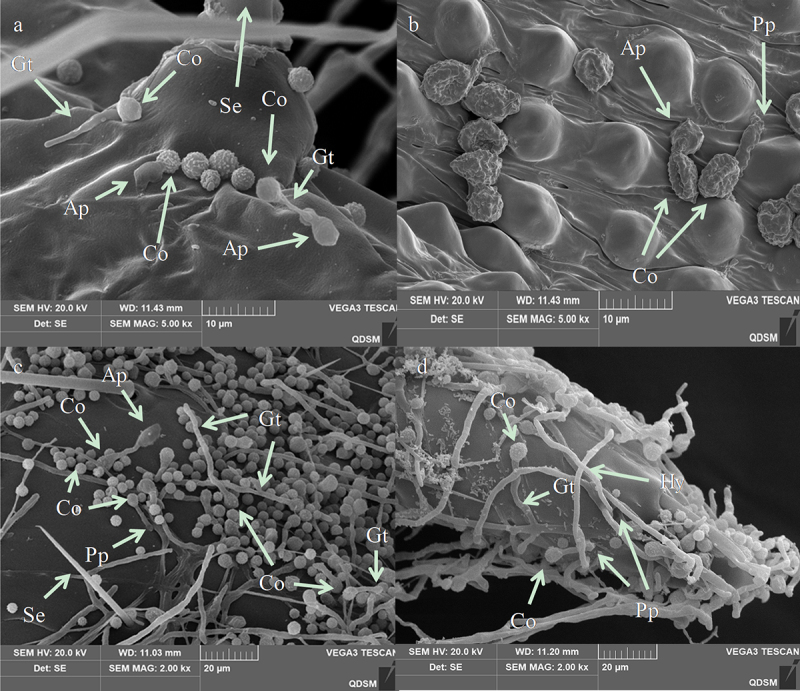
Conidial attachment and germination of *A. nomius* on the cuticle of the 3^rd^ instar larvae of *O. furnacalis* observed under a scanning electron microscope. (a) conidium (Co) attached to the setal alveolus and the germination of a few conidia with formed germ tube (gt) within 8 h and appressorium (ap) at 24 h post-inoculation; seta = se. (b) appressorium (ap) and penetration pegs (pp) formed at 36 h post-inoculation. (c and d) numerous conidia germinated to form a germ tube (gt), conidial germination rates increased, and mycelium formed on the host surface within 36 h; penetration pegs = pp, hyphae = hy.

The infection process began with the attachment of *A. nomius* conidia to the larval cuticle of the 3^rd^ instar larvae *O. furnacalis* (Figure 7). SEM analysis of infected insects showed that the conidia of *A. nomius* were firmly attached to the surface topography of both types of larval cuticle on the 3^rd^ instar larvae of *O. furnacalis*, including surfaces containing protrusions (Figure 7a), thoracic foot (Figure 7b), head capsule (Figure 7c) and setal alveolus (Figures. 7a and b).

**Figure 8.**
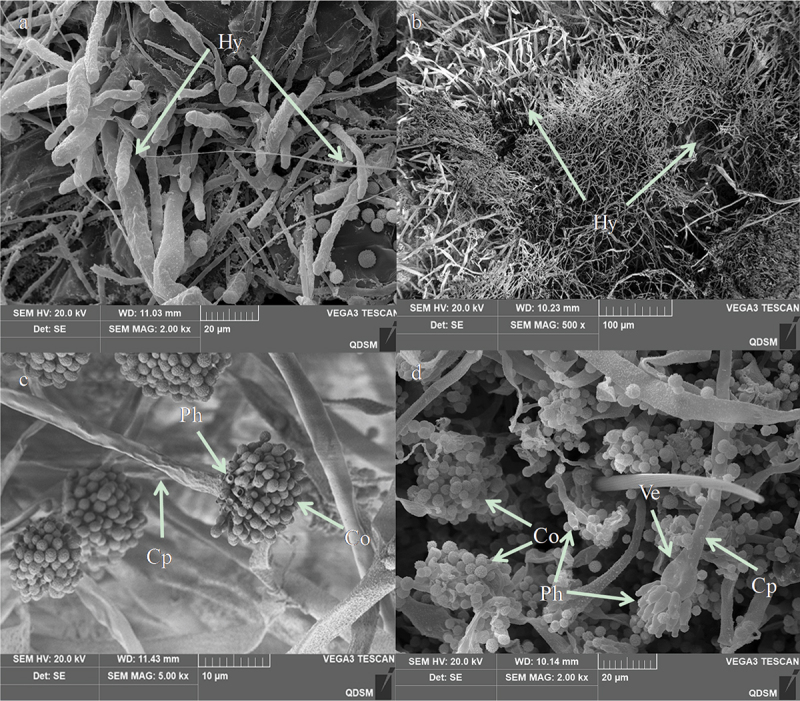
Penetration and conidia development and secondary conidiogenous structure of *A. nomius*on the cuticle surface of the 3^rd^ instar larvae of*O. furnacalis*. (a) several hyphae penetrating the body wall from the setal alveolus outward from the body at 60 h; hyphae = hy. (b) hyphae of *A. nomius*developed, branched, and formed a dense mycelial mass on the cuticle 72 h after inoculation; hyphae = hy. (c and d) secondary conidiogenous structure formation of *A. nomius*84 h after inoculation; phialides = Ph, conidiophores = cp, vesicle = ve.

Morphogenetic events of *A. nomius* were easily observed near the setal alveolus (Figure 8a), surfaces containing protrusions (Figure 8b), sites beside the setae (Figure 8c), and thoracic foot cuticle of the larvae (Figure 8d). Differentiation of conidia into a germ tube, which is a sign of *A. nomius* conidia germination, occurred within 8 h following inoculation (Figure 8). Two types of specialized infection structures, including appressoria and penetration pegs, were produced during germination (Figure 8). The germ tubes from the conidia (Figure 7c) and the penetration pegs and appressoria from the germ tubes (arrowhead in Figure 7a) were observed near the setae and on surfaces containing protrusions and the thoracic foot. Appressoria and penetration pegs were formed at the end of the germ tubes, most of which were covered by a thin amorphous mucilage layer that firmly adhered the appressoria and penetration pegs to the *O. furnacalis* integument and then penetrated through the cuticle of the 3^rd^ instar larvae of *O. furnacalis* (Figure 8). Within 36 h, conidial germination rates increased. Most germ tubes and mycelium had formed on the surfaces of the 3^rd^ instar larvae of *O. furnacalis* (Figures. 8c and d).

**Figure 9.**
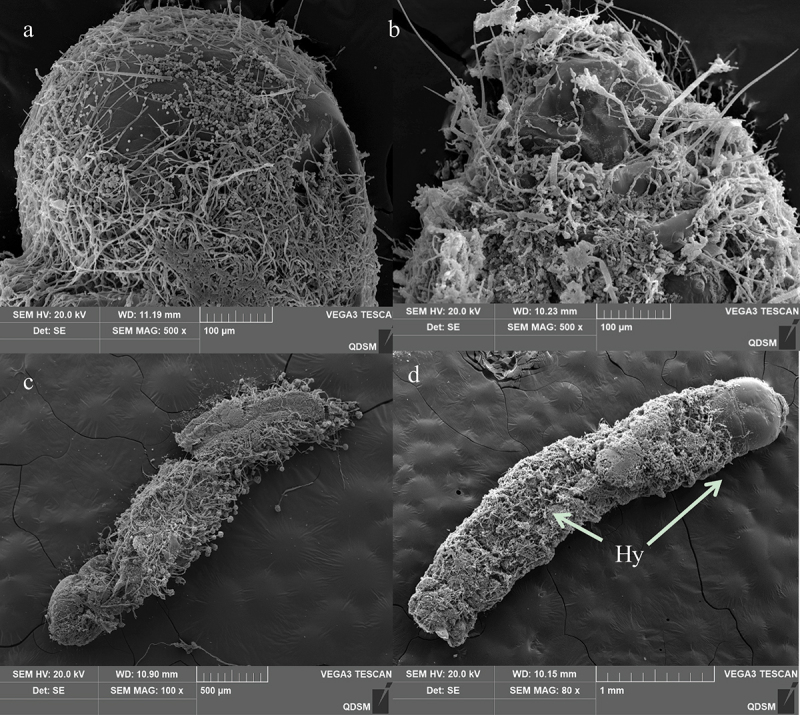
Scanning electron representative microscopy images of the 3^rd^ instar larvae of *O. furnacalis* infected with *A. nomius*(120 h post-infection). (a) head capsule with abundant conidia and mycelial growth. (b) Extensive growth of mycelia in the abdomen. (c and d) full view of a larva showing complete mycelial networking.

Hyphae extrusion from sites near the protrusions on the larval cuticle was observed at 60 h following inoculation (Figures. 9a and b). Extensive mycelial networking and a secondary conidiogenous *A. nomius* structure were noted on the cadaver 84 h after inoculation (Figures. 9c and d). After 120 h after inoculation, *A. nomius* had colonized the entire cadaver. The cadaver exhibited a mycoses appearance (Figure 9). Extensive mycelial networking and sporulation were observed on the head region (Figure 9a) and abdomen (Figure 9b). Weakly sclerotized intersegmental regions were colonized by the mycelium, which was also extruded from the surface of the cadaver (Figure 9c). The conidia of *A. nomius* were carried on vesicles (Figures. 9c and d) and conidiophores (Figure 9d), and conidiophores were observed throughout the cadaver.

### Extracellular enzyme assay and its relationship with A. nomius virulence

**Figure 10.**
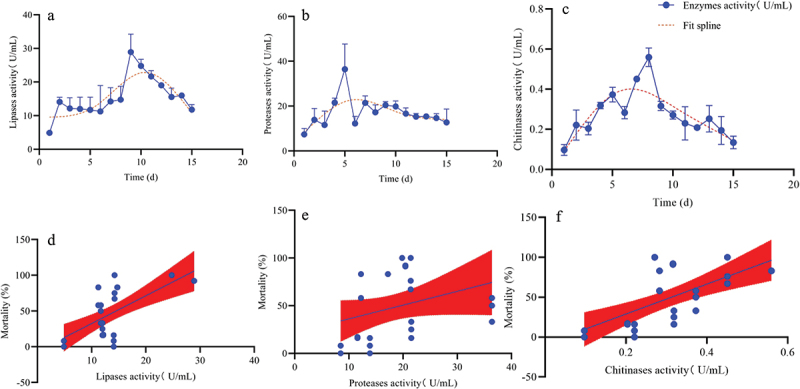
Activities of extracellular enzymes and their correlation with the virulence of *A. nomius*. (a) lipase activity, (b) protease activity, (c) chitinase activity, (d) relationship between the mortality rate of the 3^rd^ instar larvae of *O. furnacalis* and lipase activity, (e) relationship between the mortality rate of the 3^rd^ instar larvae of *O. furnacalis* and protease activity, (f) relationship between the mortality rate of 3^rd^ instar larvae of *O. furnacalis* and chitinase activity.

The lipase, protease, and chitinase activities of *A. nomius* gradually increased, peaked, and decreased slowly over the culture duration (Figure 7). The maximum lipase activity (28.92 U/mL) occurred on day 9 (Figure 10a), while those of protease (21.42 U/mL) and chitinase (0.56 U/mL) were observed on days 5 (Figure 10b) and 8 (Figure 10c), respectively. Furthermore, minimum lipase (4.87 U/mL), protease (8.49 U/mL), and chitinase (0.10 U/mL) activities were observed on day 1 (Figure 10). A strong correlation between lipase activity and the mortality rate of the 3^rd^ instar larvae of *O. furnacalis* was noted (F = 21.14, *P* = 0.0001, R^2^ = 0.8451), and a good correlation among protease and chitinase activity with mortality (F = 11.81, *P* = 0.03, R^2^ = 0.5008, F = 18.48, *P* = 0.05, R^2^ = 0.4122) was observed.

## Discussion

Although there are several commercial mycoinsecticides, it is important to investigate the use of native EPF because native isolates ensure virulence, are environmentally adapted, and provide the least non-targeted effects when used on a field scale [13]. Numerous studies have been conducted on the virulence of EPF; however, these studies have focused predominantly on certain life stages of an insect species [41–44], although the potential of EPF to infect any life stage of an insect has been confirmed [44]. The pathogenicity of EPF depends on the insect species, larval stage, and EPF strain owing to differences in the physical structure, chemical composition, and structure of the microbial community of the cuticle of each insect species [42,44,45]. As the response to insect EPF stress differs throughout their life stages [44], it is necessary to explore the mechanisms of action of indigenous and virulent EPF strains against different insect life stages to develop effective mycoinsecticides [46]. Thus, this study was designed to assess the efficacy of *A. nomius* against *O. furnacalis* at different life stages to develop an efficient fungal biopesticide to combat this maize pest in China.

The key features of a highly pathogenic EPF strain are high mortality and mycosis and low LC_50_ and LT_50_ values [2,39,47]. We compared the virulence of the *A. nomius* ACB1041 strain in *O. furnacalis* at different life stages and revealed that *A. nomius* had a significant ovicidal response in *O. furnacalis* eggs. Our results are consistent with those previous studies reporting that *M. anisopliae* and *B. bassiana* isolates significantly induced the mortality of *Spodoptera frugiperda* [30,35,44] and *Phthorimaea operculella* eggs [48]. In our study, for the larval stage, *A. nomius* provided a dose-dependent effect against *O. furnacalis*, and higher mortality and mycosis and lower LC_50_ and LT_50_ values were recorded in the younger larvae of *O. furnacalis* (1^st^, 2^nd^, and 3^rd^ instar larvae); in contrast, comparatively lower mortality and mycosis and higher LC_50_ and LT_50_ values were observed in older larvae (4^th^ and 5^th^ instar larval), pupae, and adults of *O. furnacalis*. These results, for the first time, indicate that early instar larvae (1^st^, 2^nd^, and 3^rd^ instar larvae) of *O. furnacalis* are highly susceptible to *A. nomius*; however, 3^rd^ instar larvae were more highly susceptible to *A. nomius*, which may have been attributed to differences in the physical structure and chemical composition of cuticles of *O. furnacalis* at different life stages, as well as its nutrition [29,39,44–46]. Notably, some adults that emerged from pupae infected with *A. nomius* were malformed, and the frequency of malformation was dose-dependent and increased with the spore concentration dose. This result is consistent with a previous study reporting a similar effect when *I. fumosorosea* was applied to *Spodoptera littoralis* (Boisd.) and *Cydalima perspectalis* [56].

This study showed that *A. nomius* isolates could infect *O. furnacalis* throughout its various life stages, indicating its potential use as a biological control for these pests. Although the pathogenicity of the *Aspergillus* genus to infect insects has been reported previously [2,15,17,39,49], the insecticidal activity of some *Aspergillus* species may be attributed to various secondary metabolites it contains [28]. In detail, Kaur et al. [50] detected the presence of various phenolic compounds, including gallic acid, caffeic acid, quercetin and kaempferol, by UHPLC in *A. flavus* extract. Thus, the mycoses individuals in this study may represent secondary infections of dead animals killed by secondary metabolites or toxins. At present, whether the strain ACB1041 produces mycotoxins remains unclear; thus, further studies are required to clarify this. In addition, including mortality data from ingested conidia would be useful; therefore, exploring this topic in the future for reflecting a likely transmission route in the field was also necessary. Moreover, because the 3^rd^ instar larvae of *O. furnacalis* showed the highest susceptibility to *A. nomius*, we selected them for subsequent experiments investigating the mode of action of *A. nomius* using SEM analysis. Therefore, more studies are required to explore the mechanisms underlying the highest pathogenicity of *A. nomius* against the 3^rd^ instar larvae of *O. furnacalis*.

Entomopathogenic fungi attachment and germination to the host epidermis is the first and most critical step in the infection process [24]. In our present study, extensive conidia of *A. nomius* adhered to surfaces containing protrusions, the thoracic foot, the head capsule, and the setal alveolus of the 3^rd^ instar larvae of *O. furnacalis*. Furthermore, *A. nomius* produced infection structures, including appressoria and penetration pegs, and hyphae grew during the initial phases of infection. Successful germination of fungal conidia in the host cuticle is necessary for infection [51]; these results indicated that a high conidial attachment to the larvae cuticle had occurred. These structures are important for the direct penetration of EPF into the host’s integument [52]. In addition, the key stages of host EPF infection include adhesion, germination, penetration, colonization of the hemocoel, and conidiogenesis [51]. This study preliminarily observed the infection symptoms, germination, attachment, penetration, and conidial sporulation of *A. nomius*. However, future research should be conducted to demonstrate the mechanism by which the fungus penetration and colonization of the hemocoel kill *O. furnacalis* and characterize the physiological interactions between fungi and insects as well as the comparative study of the similarities and differences between the 3rd instar larvae infected by *A. nomius* and other stages of *O. furnacalis*.

The conidia of several EPF do not attach to the head capsule of lepidopterans larvae because it is both sclerous and smooth [24]. In this study, the conidia and hyphae of *A. nomius* were evident around the head capsule of the 3^rd^ instar larvae of *O. furnacalis*, and they produced infection structures when the germ tube contacted a hard surface, which may explain the higher virulence of *A. nomius* against the 3^rd^ instar larvae of *O. furnacalis*. The cuticles of some insects contain antifungal compounds that repress germination or the further development of EPF spores, ultimately leading to infection failure [39,53]. This phenomenon could occur in the later life-cycle stages of *O. furnacalis*. Nevertheless, further research is required to explore the mechanisms underlying the lower pathogenicity of *A. nomius* in the later life-cycle stages of *O. furnacalis.*

The insecticidal mechanism underlying the pathogenicity of EPF relates to the presence of cuticle-degrading enzymes and extracellular enzymes, which are the virulence factors of many EPFs [22,54]. Our results revealed differences in the activities of extracellular enzymes with an extension in the incubation time. The highest proteases, chitinase, and lipase activity were recorded on days 5, 8, and 9, respectively. The higher protease activity indicated that *A. nomius* is capable of protein digestion in the initial stages of infection, and protease activity may then ensure the success of chitinase and lipase to feasibly penetrate the *O. furnacalis* cuticle. A similar observation was reported, in which proteases were produced during invasion before chitinases, which are important in cuticle infiltration [22]. Furthermore, in this study, *A. nomius* showed a high lipase activity, and lipases may be important for integument lipids in EPF development. Similar results have been reported in *B. bassiana* that infects *Chilo suppressalis* Walker [13]. In this study, the activities of proteases, chitinases and lipase were highly correlated with mortality, which is consistent with the results of previous studies reporting that EPF with the highest enzymatic activity resulted in higher mortality rates [53,54], and that EPF with higher protease and chitinase activities resulted in lower LC_50_ and LT_50_ values [22]. Currently, the expression genes of various extracellular enzymes of the *A. nomius* ACB1041 strain against ingests are unclear. Therefore, it is necessary not only to find the expression genes of various enzymes through genetic technology by the biotechnology method but also to express these genes in large quantities and finally achieve the effect of increasing the enzyme activity and enhancing the pathogen virulence [55,56]. Only after the above issues are clarified can we further develop this new resource and promote the application of biological pesticides.

In conclusion, investigating EPF virulence in different life stages of insect species may help determine the most appropriate insect life stage that can be targeted when using EPF as an effective biological control agent. In this study, the virulence of the *A. nomius* ACB1041 strain against the eggs, 1^st^‒5^th^ instar larvae, pupae, and adults of *O. furnacalis* was compared, and the results showed that the insecticidal ability of the *A. nomius* ACB1041 strain was higher against younger larvae of *O. furnacalis* than against older larvae, pupae, and adults. Our results also showed that the *A. nomius* ACB1041 strain has a promising biological control effect against *O. furnacalis*, highlighting its potential for development as a new biological control agent.

## Data Availability

The data used to support the findings of this study are available from the corresponding author upon request.
